# Discharge outcomes of intravenous alteplase 4.5–24 hours after symptom onset in selected patients with acute ischemic stroke: a propensity score–matched real-world study

**DOI:** 10.1186/s42466-026-00529-w

**Published:** 2026-09-04

**Authors:** Lingshe Meng, Jinfeng Yin, Yingyu Jiang, Aichun Cheng, Yuting Xiong, Xiyue Cheng, Zihan Li, Chunjuan Wang

**Affiliations:** 1 Department of Neurology, Beijing Tiantan Hospital, Capital Medical University, No. 119 South 4th Ring West Road, Fengtai District, Beijing, 100070 China; 2 China National Clinical Research Center for Neurological Diseases, Beijing, China

**Keywords:** Acute ischemic stroke, Thrombolysis, Extended time window, Real-world study

## Abstract

**Background:**

Evidence for alteplase 4.5–24 h after acute ischemic stroke (AIS) onset remains limited in routine practice. We examined associations between late-window intravenous thrombolysis (IVT) and discharge outcomes in selected patients.

**Methods:**

We analyzed China Stroke Center Alliance patients arriving 4–23.5 h after onset. Alteplase-treated patients were matched 1:1 to non-IVT patients using propensity scores. The primary outcome was modified Rankin Scale (mRS) 0–1 at discharge. Secondary and safety outcomes included mRS 0–2, registry-recorded in-hospital intracranial hemorrhage (ICH), mortality, discharge against medical advice (DAMA), and mortality or DAMA.

**Results:**

Among 137,157 eligible AIS patients, 874 received IVT and were matched to 874 non-IVT patients. Median onset-to-needle time was 6.05 h (interquartile range 5.12–10.04); 48.4%, 23.7%, 6.6%, and 21.3% were treated at 4.5-<6, 6-<9, 9-<12, and 12–24 h, respectively. IVT was associated with higher rates of mRS 0–1 (30.4% vs. 17.0%, odds ratio [OR] 2.14, 95% confidence interval [CI], 1.70–2.69, *p* < 0.001) and mRS 0–2 (65.4% vs. 58.1%; OR, 1.36; 95% CI, 1.12–1.66, *p* = 0.002). In-hospital ICH was more frequent (1.7% vs. 0.6%, OR 3.04, 95% CI, 1.10–8.39, *p* = 0.03), whereas DAMA was less frequent (5.7% vs. 8.8%, OR 0.63, 95% CI, 0.44–0.92, *p* = 0.02). Length of hospital stay, mortality, and mortality or DAMA did not differ significantly.

**Conclusions:**

Late-window alteplase was associated with better discharge function but more registry-recorded ICH in selected patients. Because symptomatic status, hemorrhage subtype, severity, and fatality were unavailable, hemorrhagic safety remains incompletely characterized. These observational findings require cautious interpretation.

**Supplementary Information:**

The online version contains supplementary material available at https://doi.org/10.1186/s42466-026-00529-w.

## Key summary points

**Why carry out this study?**

Evidence from randomized trials suggests that intravenous thrombolysis (IVT) beyond 4.5 hours after acute ischemic stroke (AIS) onset may be beneficial in carefully selected patients, usually with imaging-based selection.

However, real-world data on alteplase administered 4.5–24 hours after symptom onset remain limited, particularly in large Chinese stroke cohorts.

**What was learned from the study?**

In this propensity score-matched real-world study, late-window IVT was associated with higher rates of favorable functional outcomes at discharge among selected patients with AIS, but registry-recorded in-hospital intracranial hemorrhage was more frequent. In-hospital mortality, mortality or discharge against medical advice, and length of hospital stay did not differ significantly between groups. However, the registry did not distinguish symptomatic from asymptomatic ICH or identify severe or fatal hemorrhage; therefore, the safety findings remain incomplete and should be interpreted cautiously.

**Supplementary Information:**

The online version contains supplementary material available at https://doi.org/10.1186/s42466-026-00529-w.

## Introduction

Since the publication of the NINDS and the ECASS III trials [1, 2], intravenous thrombolysis (IVT) with alteplase has been recommended for eligible patients with acute ischemic stroke (AIS) within 4.5 h after symptom onset [3, 4]. However, a substantial proportion of AIS patients arrive beyond this conventional treatment window, with only approximately 30% reaching the hospital within 4.5 h [5]. Therefore, identifying patients who may benefit from alteplase beyond 4.5 h has become an important area of stroke research.

Earlier trials evaluating alteplase beyond the standard time window, including ECASS II and ATLANTIS, did not establish a clear overall benefit [6–8]. More recent trials have shifted the focus from a purely time-based approach to patient selection based on imaging or specific clinical profiles. EXTEND showed that alteplase could improve functional outcomes up to 9 h after onset in patients selected by perfusion imaging [9]. WAKE-UP supported magnetic resonance imaging (MRI)-guided thrombolysis in patients with unknown time of onset and a diffusion-weighted imaging–fluid-attenuated inversion recovery mismatch [10]. More recently, TRACE-Ⅲ and HOPE extended thrombolysis research to the 4.5–24-hour window in selected patients, using tenecteplase and alteplase, respectively [11, 12]. EXPECTS further suggested a potential role for alteplase in selected patients with posterior circulation stroke [13]. Together, these studies indicate that late-window thrombolysis may be feasible in carefully selected patients, but the evidence is highly dependent on patient-selection criteria.

Although these trials provide important evidence for thrombolysis beyond 4.5 h in selected patients, their findings may not fully reflect routine clinical practice, where imaging resources, treatment protocols, and patient characteristics can vary substantially across centers. Real-world evidence on alteplase administered 4.5–24 h after symptom onset remains limited, particularly in large Chinese stroke cohorts. Therefore, using data from a nationwide registry, we investigated the association between alteplase administered within 4.5–24 h after symptom onset and in-hospital outcomes among selected patients with AIS.

## Methods

### Data source and study population

The China Stroke Center Alliance (CSCA) is a national, hospital-based, multicenter registry and quality-improvement initiative launched in 2015. It was designed to support the development of stroke centers, promote guideline-based stroke care, and improve the quality of care and clinical outcomes for patients with cerebrovascular diseases [14]. 

The registry continuously enrolled patients aged 18 years or older who were admitted within 7 days after symptom onset and diagnosed by computed tomography (CT) or MRI with AIS, transient ischemic attack, intracerebral hemorrhage, or subarachnoid hemorrhage. Collected variables included demographics, vascular risk factors, medical and medication history, hospital presentation, initial neurological status, acute treatments, reperfusion strategies, in-hospital complications, and discharge outcomes.

Each participating hospital obtained institutional review board approval or a waiver of individual informed consent for data collection in the CSCA registry, as appropriate. The data coordinating center is located at Beijing Tiantan hospital, Capital Medical University.

For the present analysis, we identified patients with AIS admitted between January 1, 2019, and December 14, 2022, who had an onset-to-door time (ODT) of 4–23.5 h. This ODT range was used to define a common extended-window source population for both treated and untreated patients and to allow patients in the IVT group to receive alteplase within 4.5–24 h after symptom onset. Patients were excluded if they had a prestroke modified Rankin Scale (mRS) score > 2, received endovascular thrombectomy (EVT), were treated with urokinase, had missing key treatment-allocation data, or had documented contraindications to IVT other than time window, including intracranial hemorrhage or persistent blood pressure ≥ 185/110 mmHg despite antihypertensive treatment [3]. 

Patients who received intravenous alteplase 4.5–24 h after symptom onset were assigned to the IVT group. Patients who did not receive any thrombolytic agent were considered eligible for the non-IVT group and were matched to IVT-treated patients using 1:1 propensity score matching (PSM) as described below. Because onset-to-needle time (ONT) was defined only for patients receiving IVT, between-group time-window analyses were based on onset-to-door time (ODT). Within the IVT group, ONT was summarized descriptively using the median, interquartile range, range, and four clinically interpretable categories: 4.5-<6, 6-<9, 9-<12, and 12–24 h after symptom onset. No comparative treatment-effect analyses were performed according to ONT category.

The CSCA registry did not record detailed imaging-selection variables used for late-window thrombolysis decisions, including multimodal imaging criteria, infarct core or penumbral status, vessel occlusion status, collateral status, or infarct location. Therefore, these variables could not be incorporated into PSM, subgroup analyses, or interpretation of treatment selection.

### Outcomes

The primary outcome was favorable functional outcome at discharge, defined as an mRS score of 0–1. Because 90-day follow-up data were not available in the CSCA registry, functional status was assessed at discharge. Secondary outcomes included functional independence (defined as mRS 0–2) at discharge, independent ambulation (defined as mRS 0–3) at discharge, discharge directly home, and length of hospital stay.

Safety outcomes included registry-recorded in-hospital ICH, in-hospital mortality, discharge against medical advice (DAMA), and the composite outcome of in-hospital mortality or DAMA. Registry-recorded in-hospital ICH was defined according to the CSCA variable indicating whether any intracranial hemorrhage occurred during hospitalization. The registry did not capture sufficient information to distinguish symptomatic from asymptomatic ICH, classify hemorrhage according to ECASS or SITS criteria, identify hemorrhage subtype, assess neurological deterioration attributable to hemorrhage, or determine whether an ICH event was fatal. Accordingly, this endpoint represents any registry-recorded in-hospital ICH and should not be interpreted as symptomatic ICH.

Prespecified subgroup analyses for the primary outcome were performed according to age (< 80 vs. ≥ 80 years), sex, admission National Institutes of Health Stroke Scale (NIHSS) score (< 4 vs. ≥ 4), and ODT category (4–5.5, 5.5–8.5, 8.5–11.5, and 11.5–23.5 h).

### Statistical analysis

Categorical variables are reported as frequencies and percentages. Continuous variables are presented as means with standard deviations or medians with interquartile ranges (IQR), according to their distribution. Statistical comparisons were performed using the chi-squared test or Fisher’s exact test for binary variables, as well as the Student’s t-test or Mann-Whitney U test for continuous variables.

The primary analysis was conducted in patients with available admission NIHSS scores. To evaluate potential differences related to missing admission NIHSS data, baseline characteristics were compared between patients with and without available admission NIHSS scores before the analysis.

PSM was used to balance measured baseline characteristics between the IVT and non-IVT groups [15]. Patients in the IVT group were matched 1:1 to patients in the non-IVT group. Covariate balance after matching was assessed using standardized mean differences (SMDs), with an absolute SMD < 0.10 indicating acceptable balance.

Binary outcomes were compared between groups using logistic regression models and are reported as odds ratios (ORs) with 95% confidence intervals (CIs). Length of hospital stay was analyzed as a continuous variable.

As a sensitivity analysis, multiple imputation was performed to assess the influence of missing admission NIHSS scores. Missing NIHSS values were imputed using 10 imputed datasets. The multivariable logistic regression analyses were repeated within each imputed dataset using the same covariates as in the main analysis, and pooled estimates were obtained according to Rubin’s rules.

A two-sided P value < 0.05 was considered statistically significant. Patients with missing outcome data (such as mRS score at discharge, death or DAMA) were excluded from the corresponding outcome analysis. All analyses were conducted using the R software, version 4.3.1.

## Results

### Baseline characteristics

**Fig. 1:**
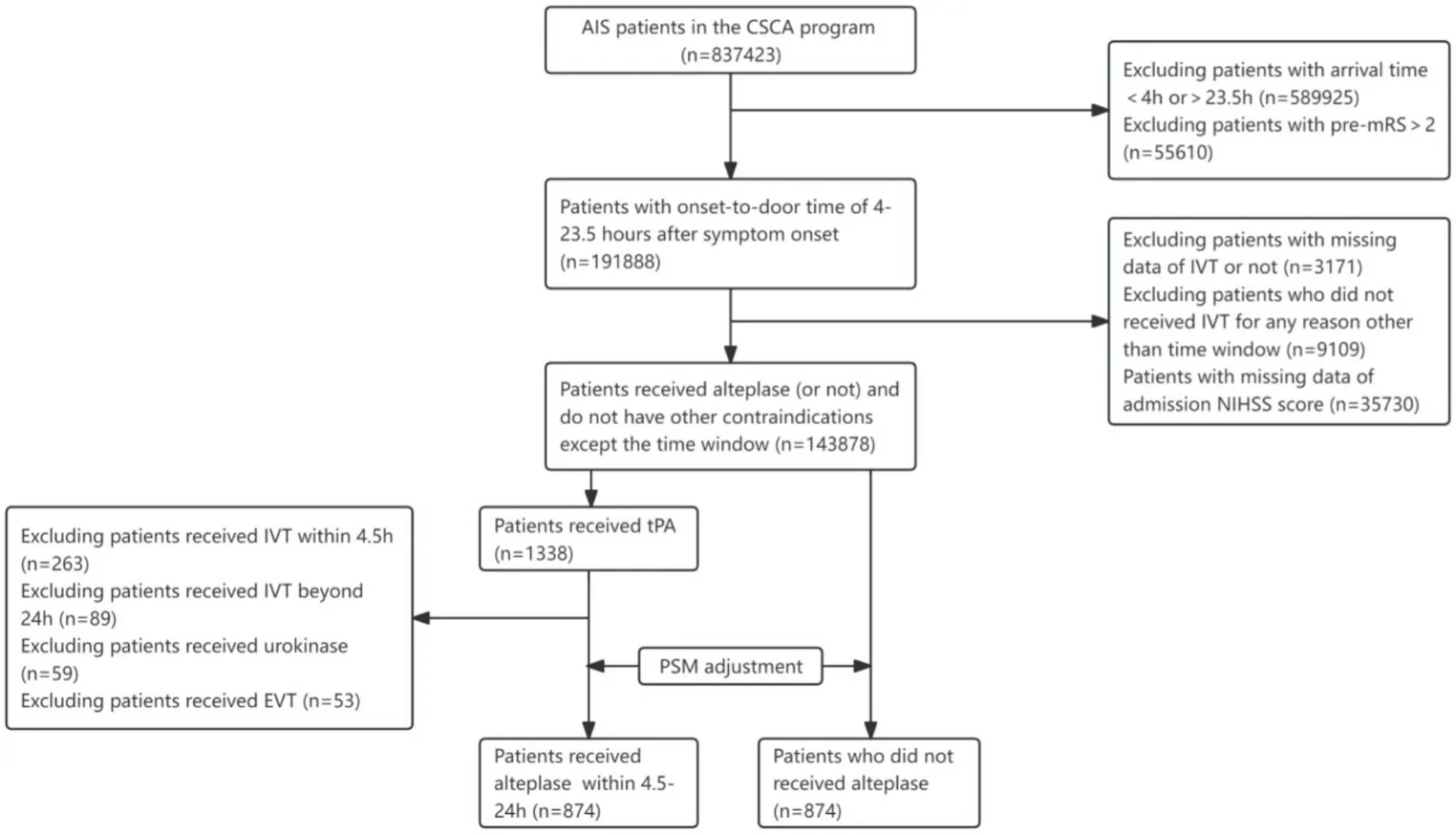
Flowchart of patient selection. AIS, Acute Ischemic Stroke; CSCA, China Stroke Center Alliance; IVT, Intravenous Thrombolysis; NIHSS, National Institutes of Health Stroke Scale; ODT, Onset-to-Door Time; PSM, Propensity Score Matching

Among 837,423 patients in the CSCA registry during the study period, 172,887 met the initial eligibility criteria. After excluding 35,730 patients with missing admission NIHSS scores, 137,157 patients with AIS and an ODT of 4–23.5 h were included in the primary complete-case analysis (Fig. 1). Baseline characteristics were generally similar between patients with and without available admission NIHSS scores, although the proportion treated at tertiary hospitals differed between the two groups (Table S1).

Before matching, 874 patients received intravenous alteplase 4.5–24 h after symptom onset, whereas 136,283 patients did not receive IVT. The IVT group differed from the non-IVT group in several baseline characteristics, particularly admission NIHSS score, ODT, and hospital level (Table S2). After 1:1 propensity score matching, 874 IVT-treated patients were matched to 874 non-IVT patients. In the IVT group, the median onset-to-needle time was 6.05 h (IQR 5.12–10.04, range 4.50–23.98). Of the 874 IVT-treated patients, 423 (48.4%) received alteplase at 4.5-<6 h, 207 (23.7%) at 6-<9 h, 58 (6.6%) at 9-<12 h, and 186 (21.3%) at 12–24 h after symptom onset (Table S4).

In the matched cohort, the median age was 65 years, 65.4% of patients were male, 57.1% had hypertension, 19.3% had diabetes mellitus, and the median admission NIHSS score was 4. Baseline characteristics were all balanced between the IVT and non-IVT groups after matching, with all absolute standardized mean differences < 0.10 (Table 1).

**Table 1:** Baseline characteristics of the propensity score-matched cohort

Variables	Non-IVT (*n* = 874)	IVT (*n* = 874)	*P* value	SMD
**Age**,** years**,** median (IQR)**	65.0 (55.0, 73.0)	65.0 (56.0, 73.0)	0.60	0.03
**Male**,** n**,** (%)**	562 (64.3)	581 (66.5)	0.37	0.05
**BMI**,** median (IQR)**	23.4 (21.5, 25.4)	23.4 (21.4, 25.7)	0.79	0.04
**History of smoking**,** n**,** (%)**	333 (38.1)	340 (38.9)	0.77	0.02
**History of drinking**,** n**,** (%)**	239 (27.3)	228 (26.1)	0.59	0.03
**Hypertension**,** n**,** (%)**	499 (57.1)	499 (57.1)	1.00	< 0.001
**Diabetes mellitus**,** n**,** (%)**	161 (18.4)	176 (20.1)	0.40	0.04
**Dyslipidemia**,** n**,** (%)**	60 (6.9)	49 (5.6)	0.32	0.05
**Atrial fibrillation**,** n**,** (%)**	50 (5.7)	49 (5.6)	1.00	0.005
**Prior TIA**,** n**,** (%)**	11 (1.3)	11 (1.3)	1.00	< 0.001
**Prior ischemic stroke**,** n**,** (%)**	170 (19.5)	186 (21.3)	0.37	0.05
**Prior peripheral vascular disease**,** n**,** (%)**	3 (0.3)	5 (0.6)	0.72	0.03
**Prior myocardial infarction**,** n**,** (%)**	18 (2.1)	13 (1.5)	0.47	0.04
**NIHSS score at admission**,** median (IQR)**	4.5 (3.0, 9.0)	4.0 (2.0, 8.0)	0.24	0.08
**Onset-to-door time**,** median (IQR)**	6.1 (5.0, 8.6)	5.2 (4.5, 8.3)	0.24	0.08
**Treated at a tertiary hospital**,** n**,** (%)**	546 (62.5)	534 (61.1)	0.59	0.03

BMI, Body Mass Index; TIA, Transient Ischemic Attack; NIHSS, National Institutes of Health Stroke Scale; SMD, Standardized Mean Difference; PSM, Propensity Score Matching

### In-hospital outcomes

**Fig. 2:**
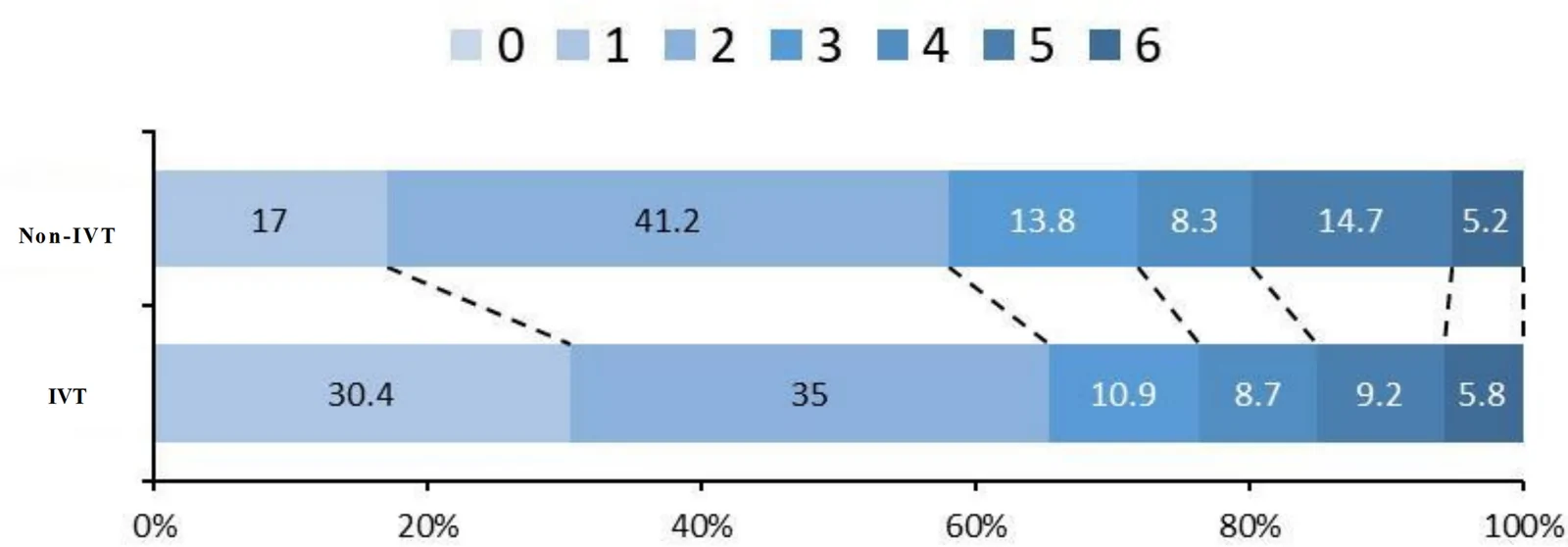
Distribution of modified Rankin Scale score in the propensity score-matched cohort

Efficacy and safety outcomes in the propensity score-matched cohort are shown in Table 2; Fig. 2.

**Table 2:** Discharge and in-hospital outcomes in the propensity score-matched cohort

Outcomes	Non-IVT (*n* = 874)	IVT (*n* = 874)	Effect estimate (95% CI)	*P* value
**Primary outcome**				
mRS 0–1 at discharge*	148 (17.0)	266 (30.4)	2.14 (1.70, 2.69)	< 0.001
**Secondary outcomes**				
mRS 0–2 at discharge*	507 (58.1)	572 (65.4)	1.36 (1.12, 1.66)	0.002
Independent ambulation at discharge*	627 (71.9)	667 (76.3)	1.26 (1.02, 1.56)	0.04
Discharge directly home*	768 (87.9)	779 (89.1)	1.13 (0.84, 1.52)	0.41
**Length of hospital stay**,** days**	11.00 (7.00, 14.00)	10.00 (7.00, 14.00)	−0.52 (−1.08, 0.05)	0.08
**Safety outcomes**				
** Registry-recorded in-hospital ICH**	5 (0.6)	15 (1.7)	3.04 (1.10, 8.39)	0.03
** In-hospital mortality**	45 (5.1)	51 (5.8)	1.14 (0.76, 1.72)	0.53
DAMA*	76 (8.8)	50 (5.7)	0.63 (0.44, 0.92)	0.02
In-hospital mortality or DAMA*	108 (12.4)	87 (10.0)	0.78 (0.58, 1.01)	0.11

Odds ratios are reported for binary outcomes, and β coefficients are reported for length of hospital stay

* Data were missing for discharge mRS in 202 patients, DAMA in 385 patients, and in-hospital mortality or DAMA in 147 patients

Registry-recorded in-hospital ICH represented any intracranial hemorrhage recorded during hospitalization. Information on symptomatic status, ECASS/SITS classification, hemorrhage subtype, hemorrhage-related neurological deterioration, and fatal ICH was unavailable

ICH, Intracranial Hemorrhage; DAMA, Discharge Against Medical Advice; IVT, Intravenous Thrombolysis; PSM, Propensity Score Matching; OR, Odds Ratio; CI, Confidence Interval

The primary outcome of favorable functional outcome at discharge, defined as mRS 0–1, occurred in 266 patients (30.4%) in the IVT group and 148 patients (17.0%) in the non-IVT group. IVT was associated with a higher rate of mRS 0–1 at discharge (odds ratio [OR] 2.14, 95% confidence interval [CI] 1.70–2.69, *p* < 0.001).

Secondary discharge outcomes showed a similar pattern. Functional independence at discharge, defined as mRS 0–2, was observed in 572 patients (65.4%) in the IVT group and 507 patients (58.1%) in the non-IVT group (OR 1.36, 95% CI 1.12–1.66, *p* = 0.002). Independent ambulation at discharge, defined as mRS 0–3, occurred in 667 patients (76.3%) and 627 patients (71.9%), respectively (OR 1.26, 95% CI 1.02–1.56, *p* = 0.04). The rate of discharge directly home did not differ significantly between the IVT and non-IVT groups (OR 1.13, 95% CI 0.84–1.52, *p* = 0.41). Length of hospital stay was not significantly different between groups, with a median of 10.00 days (IQR, 7.00–14.00) in the IVT group and 11.00 days (IQR, 7.00–14.00) in the non-IVT group (β, −0.52; 95% CI, −1.08 to 0.05; *P* = 0.075).

For safety outcomes, registry-recorded in-hospital ICH was more frequent in the IVT group than in the non-IVT group (1.7% vs. 0.6%; OR 3.04, 95% CI 1.10–8.39, *p* = 0.03). DAMA was less frequent in the IVT group (5.7% vs. 8.8%; OR 0.63, 95% CI 0.44–0.92, *p* = 0.02). In-hospital mortality did not differ significantly between groups (5.8% vs. 5.1%, OR 1.14, 95% CI 0.76–1.72, *p* = 0.53), nor did the composite outcome of in-hospital mortality or DAMA (10.0% vs. 12.4%, OR 0.78, 95% CI 0.58–1.01, *p* = 0.11).

### Subgroup analyses

**Fig. 3:**
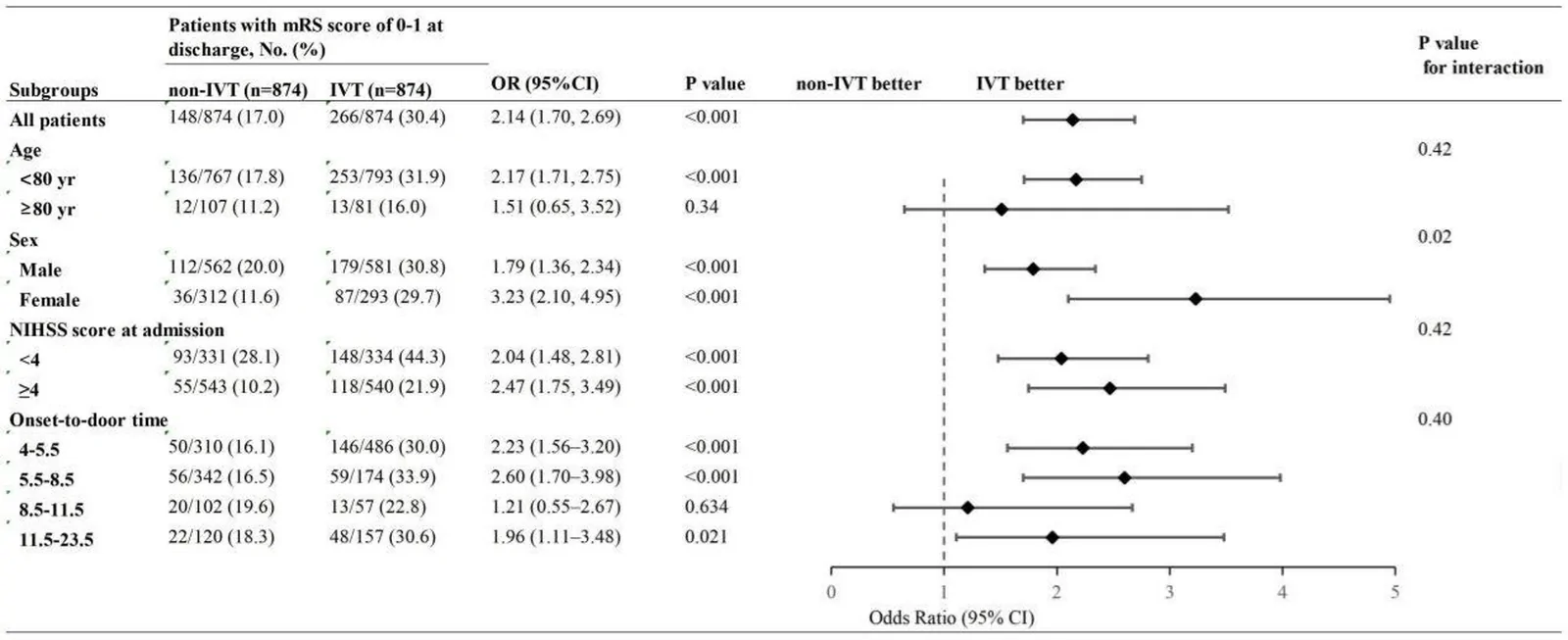
Subgroup analyses for favorable functional outcome at discharge. Odds ratios were estimated using logistic regression models within the matched cohort. ODT categories were used for comparative subgroup analyses because ONT was defined only for patients receiving IVT. ONT was summarized descriptively within the IVT group and was not used for between-group treatment-effect estimation. CI, confidence interval; IVT, intravenous thrombolysis; NIHSS, National Institutes of Health Stroke Scale; ODT, onset-to-door time; OR, odds ratio. For the onset-to-door time subgroup analysis, P for trend across increasing onset-to-door time categories was 0.49

Subgroup analyses for the primary outcome are shown in Fig. 3.

The association between IVT and favorable functional outcome at discharge was generally consistent across age groups and admission NIHSS strata. Among patients aged < 80 years, mRS 0–1 at discharge occurred in 253 of 793 patients (31.9%) in the IVT group and 136 of 767 patients (17.8%) in the non-IVT group (OR 2.17, 95% CI 1.71–2.75, *P* < 0.001). Among patients aged ≥ 80 years, the corresponding rates were 16.0% and 11.2%, respectively (OR 1.51, 95% CI 0.65–3.52, *P* = 0.34, P for interaction = 0.42).

When stratified by admission NIHSS score, IVT was associated with higher rates of mRS 0–1 both in patients with NIHSS < 4 (44.3% vs. 28.1%, OR 2.04, 95% CI 1.48–2.81, *P* < 0.001) and in those with NIHSS ≥ 4 (21.9% vs. 10.2%, OR 2.47, 95% CI 1.75–3.49, *P* < 0.001), with no significant interaction by NIHSS category (P for interaction = 0.42). A treatment-by-sex interaction was observed (P for interaction = 0.02), with a larger point estimate in women (OR 3.23, 95% CI 2.10–4.95) than in men (OR 1.79, 95% CI 1.36–2.34).

In the analysis stratified by ODT category, IVT was associated with higher rates of mRS 0–1 at discharge in the 4–5.5-hour group (30.0% vs. 16.1%, OR 2.23, 95% CI 1.56–3.20, *P* < 0.001), the 5.5–8.5-hour group (33.9% vs. 16.5%, OR 2.60, 95% CI 1.70–3.98, *P* < 0.001), and the 11.5–23.5-hour group (30.6% vs. 18.3%, OR 1.96, 95% CI 1.11–3.48, *P* = 0.021). In the 8.5–11.5-hour group, the difference was not statistically significant (22.8% vs. 19.6%, OR 1.21, 95% CI 0.55–2.67, *P* = 0.634). There was no significant interaction across ODT categories (P for interaction = 0.395), and no clear trend in the association across increasing ODT categories was observed (P for trend = 0.489).

### Sensitivity analyses

The sensitivity analysis using multiple imputation for missing admission NIHSS scores is shown in Table S3.

The association between IVT and favorable functional outcome at discharge remained significant (OR 1.09, 95% CI 1.06–1.12; *P* < 0.001). Similar findings were observed for mRS 0–2 at discharge (OR 1.04, 95% CI 1.01–1.06; *P* = 0.01), whereas the association with independent ambulation defined as mRS 0–3 was attenuated and no longer statistically significant (OR 1.01, 95% CI 0.99–1.03; *P* = 0.17). Registry-recorded in-hospital ICH remained more frequent in the IVT group, while no significant association was observed for in-hospital mortality. DAMA remained less frequent after IVT. Overall, the imputed analysis showed broadly similar directions to the primary matched analysis, although some secondary discharge outcomes were attenuated.

## Discussion

In this national, real-world registry study of selected patients with AIS presenting beyond the conventional 4.5-hour time window, intravenous alteplase administered 4.5–24 h after symptom onset was associated with more favorable functional outcomes at discharge after propensity score matching. The associations were observed across several discharge functional measures, including mRS 0–1, mRS 0–2, and independent ambulation defined as mRS 0–3. Registry-recorded in-hospital ICH was more frequent in the IVT group, whereas no statistically significant between-group differences were observed in in-hospital mortality, the composite outcome of in-hospital mortality or DAMA, or length of hospital stay. Because the registry did not permit classification of symptomatic, severe, or fatal ICH, these findings do not provide a complete assessment of hemorrhagic safety. These findings suggest that late-window alteplase use in routine practice may be associated with improved short-term discharge outcomes in clinically selected patients, but the observational nature of the study and the lack of detailed imaging-selection data require cautious interpretation.

The evidence base for thrombolysis beyond 4.5 h has evolved from a purely time-based paradigm toward treatment decisions guided by tissue status, imaging profiles, or specific clinical presentations. EXTEND and WAKE-UP supported alteplase use beyond standard time windows in imaging-selected patients, while more recent trials such as TRACE-Ⅲ and HOPE evaluated thrombolysis in the 4.5–24-hour window in carefully selected patients [9–12]. EXPECTS further provided evidence for alteplase in selected patients with posterior circulation stroke [13]. These trials established the importance of patient selection in late-window thrombolysis. In contrast, the present study reflects real-world clinical practice across a large national registry, in which treatment decisions were made under heterogeneous institutional conditions rather than within the standardized framework of a randomized trial.

The real-world context is important because imaging resources, workflow efficiency, physician experience, and institutional protocols may vary substantially across hospitals. Recent real-world experience has similarly shown that intravenous thrombolysis beyond the conventional 4.5-hour window is implemented in routine practice using center-specific clinical and imaging-selection protocols [16]. In such settings, patients who receive alteplase beyond 4.5 h are likely to represent a highly selected subgroup judged by treating physicians to have an acceptable risk-benefit profile. Therefore, our findings should not be interpreted as evidence supporting generalized alteplase use for all AIS patients presenting within 4.5–24 h. Rather, they indicate that, among patients selected for treatment in routine clinical practice, late-window IVT was associated with more favorable discharge functional outcomes. This distinction is particularly important because the CSCA registry did not record detailed imaging-selection variables, including multimodal imaging criteria, infarct core or penumbral status, vessel occlusion status, collateral status, or infarct location. The potential relevance of such information is supported by recent multimodal imaging data showing that clinically mild stroke may still be accompanied by substantial perfusion-defined tissue at risk, highlighting the additional information provided by advanced imaging beyond clinical severity alone [17]. 

Regarding safety, registry-recorded in-hospital ICH was more frequent among patients treated with IVT. This association should be interpreted cautiously because only 20 ICH events occurred in the matched cohort, resulting in an imprecise effect estimate with a wide confidence interval. More importantly, the registry did not distinguish symptomatic from asymptomatic ICH, apply ECASS or SITS hemorrhage classifications, identify hemorrhage subtypes, assess hemorrhage-related neurological deterioration, or determine whether an ICH event was fatal. This distinction is important when interpreting hemorrhagic outcomes across real-world IVT studies, as recent cohorts have separately adjudicated any ICH and symptomatic ICH using standardized criteria such as SITS-MOST [18]. Although no statistically significant between-group differences were observed in in-hospital mortality or the composite outcome of mortality or DAMA, the limited number of events and the absence of detailed hemorrhage adjudication preclude conclusions regarding symptomatic, severe, or fatal bleeding risk. The relatively low absolute rate of registry-recorded ICH should also not be directly compared with hemorrhage rates reported in randomized late-window thrombolysis trials, given differences in endpoint definitions, event ascertainment, baseline stroke severity, and patient-selection procedures. Accordingly, the present safety findings should be regarded as incomplete registry-based observations rather than evidence establishing the hemorrhagic safety of late-window alteplase.

The use of discharge mRS as the primary functional outcome is another important consideration. Discharge outcomes provide useful short-term information in real-world registry studies because they reflect early neurological status and short-term care trajectories. Nevertheless, they are not equivalent to the standard 90-day functional outcomes used in randomized stroke trials and may be influenced by length of stay, discharge destination, rehabilitation access, and local discharge practices. In the present analysis, length of hospital stay did not differ significantly between the IVT and non-IVT groups, and secondary discharge outcomes, including mRS 0–2 and independent ambulation defined as mRS 0–3, showed similar directions of association, while the composite outcome of mortality or DAMA did not differ significantly between groups. Even so, longer-term follow-up remains necessary to determine whether the observed discharge associations translate into sustained functional benefit.

The descriptive ONT distribution showed that approximately half of the IVT-treated patients received alteplase within 4.5-<6 h after symptom onset, whereas approximately one fifth were treated at 12–24 h. These data provide additional information on the timing of alteplase administration in clinical practice. However, because ONT is not defined for untreated patients, the ONT categories could not be used for between-group treatment-effect estimation or interaction analyses. The comparative time-window analyses therefore remained based on ODT.

In subgroup analyses, the association between IVT and favorable functional outcome at discharge was generally consistent across age groups and admission NIHSS strata. The more granular ODT analysis did not show a significant treatment-by-time-window interaction or a clear trend across increasing ODT categories, although the 8.5–11.5-hour subgroup did not reach statistical significance. These subgroup findings should be interpreted cautiously because subgroup analyses may be underpowered and affected by clinical heterogeneity within each time window. A treatment-by-sex interaction was observed, with a larger point estimate among women than men. However, given the exploratory nature of subgroup analyses, the absence of adjustment for multiple comparisons, and the potential influence of unmeasured confounding, this finding should be considered hypothesis-generating rather than evidence for sex-specific treatment selection.

This study has several strengths. It used data from a large national stroke registry and focused on alteplase treatment in a real-world extended-window population. PSM was applied to balance measured baseline characteristics between treated and untreated patients. The sensitivity analysis using multiple imputation for missing admission NIHSS scores showed that the association with the primary discharge outcome remained significant and that most secondary and safety outcomes had directions broadly similar to the primary matched analysis. However, the association with independent ambulation was attenuated, supporting a cautious interpretation of secondary discharge outcomes. These features provide complementary real-world context to randomized trials by describing outcomes among patients treated in routine clinical settings.

Several limitations should be acknowledged. First, detailed imaging-selection data were unavailable, including the imaging modality used for treatment selection, multimodal imaging criteria, infarct core or penumbral status, vessel occlusion status, collateral status, and infarct location. Therefore, we could not determine how patients were selected for late-window IVT or whether specific imaging profiles modified the observed associations. Second, despite PSM, this observational study remains subject to residual confounding and treatment-selection bias. Important factors such as stroke mechanism, antithrombotic use, contraindication details, center-level protocols, imaging capability, and physician decision-making were not fully captured. Third, admission NIHSS scores were missing in a substantial number of otherwise eligible patients. Although multiple imputation yielded broadly similar findings for the primary outcome, selection bias related to missing NIHSS data cannot be excluded. Fourth, comparative time-window analyses were based primarily on ODT rather than ONT because ONT was defined only for IVT-treated patients. Although the ONT distribution was described within the IVT group, we could not determine whether the observed association varied according to ONT or in-hospital treatment delay. Fifth, functional outcomes were assessed only at discharge because 90-day follow-up data were unavailable. Sixth, in-hospital ICH was based on a registry-recorded variable and could not be classified as symptomatic or asymptomatic ICH, ECASS- or SITS-defined hemorrhage, specific hemorrhage subtype, hemorrhage associated with neurological deterioration, or fatal ICH. Consequently, the study cannot provide a definitive assessment of clinically relevant hemorrhagic safety. Finally, the study was conducted in Chinese hospitals participating in the CSCA registry, which may limit generalizability to other healthcare systems and populations.

## Conclusions

In this national real-world registry study of selected patients with AIS, intravenous alteplase administered 4.5–24 h after symptom onset was associated with more favorable functional outcomes at discharge, including mRS 0–1 and mRS 0–2. Registry-recorded in-hospital ICH was more frequent in the IVT group. However, because symptomatic status, hemorrhage severity, hemorrhage subtype, and fatality were unavailable, the study cannot provide a definitive assessment of hemorrhagic safety. These observational associations should be interpreted cautiously in view of potential treatment-selection bias, residual confounding, unavailable imaging-selection data, the reliance on ODT for comparative timing analyses, and the absence of 90-day functional outcomes.

## Supplementary Information

Supplementary Material 1

## Data Availability

The data that supports the findings of the current study are available from the corresponding author upon reasonable request.
