# Supplementary material for: Discharge outcomes of intravenous alteplase 4.5–24 hours after symptom onset in selected patients with acute ischemic stroke: a propensity score–matched real-world study
Source: Neurol Res Pract. 2026 Sep 4;8(1):74. doi: 10.1186/s42466-026-00529-w (PMC13545781; doi:10.1186/s42466-026-00529-w)
Supplement: Supplementary file 1 [file 42466_2026_529_MOESM1_ESM.docx]

**Cover page**

**Discharge Outcomes of Intravenous Alteplase 4.5–24 Hours after Symptom Onset in Selected Patients With Acute Ischemic Stroke: A Propensity Score–Matched Real-World Study**

Lingshe Meng, MD1,2; Jinfeng Yin, MD2; Yingyu Jiang, MD2; Aichun Cheng, MD1,2; Yuting Xiong, MD1,2; Xiyue Cheng, MD1,2; Zihan Li, MD1,2; Chunjuan Wang, MD, PhD 1,2

1 Department of Neurology, Beijing Tiantan Hospital, Capital Medical University, Beijing, China;

2 China National Clinical Research Center for Neurological Diseases, Beijing, China;

**Correspondence to:**

Chunjuan Wang

Department of Neurology, Beijing Tiantan Hospital, Capital Medical University, No. 119 South 4th Ring West Road, Fengtai District, Beijing 100070, China

Email: [wangchunjuan@](mailto:wangchunjuan@ncrcnd.org.cn)[ncrcnd.org.cn](mailto:wangchunjuan@ncrcnd.org.cn)

**Table S1. Baseline characteristics of patients with and without available admission NIHSS scores.**

| **Variables** | **Admission NIHSS available**  **(n=137157)** | **Admission NIHSS** **missing**  **(n=35730)** | **SMD** |
| --- | --- | --- | --- |
| **Age, years, median (IQR)** | 66.0 (57.0, 74.0) | 67.0 (58.0, 75.0) | 0.04 |
| **Male, n, (%)** | 88620 (64.6) | 22486 (62.9) | 0.04 |
| **BMI, median (IQR)** | 23.9 (22.0, 25.8) | 23.9 (22.0, 25.7) | 0.01 |
| **History of smoking, n, (%)** | 49052 (35.8) | 12474 (34.9) | 0.02 |
| **History of drinking, n, (%)** | 30352 (22.1) | 7831 (21.9) | 0.01 |
| **Hypertension, n, (%)** | 87015 (63.4) | 22383 (62.6) | 0.02 |
| **Diabetes mellitus, n, (%)** | 29577 (21.6) | 7431 (20.8) | 0.02 |
| **Dyslipidemia, n, (%)** | 7133 (5.2) | 1586 (4.4) | 0.04 |
| **Atrial fibrillation, n, (%)** | 4829 (3.5) | 1175 (3.3) | 0.01 |
| **Prior TIA, n, (%)** | 1216 (0.9) | 308 (0.9) | <0.01 |
| **Prior ischemic stroke, n, (%)** | 35503 (25.9) | 8679 (24.3) | 0.04 |
| **Prior peripheral vascular disease, n, (%)** | 1285 (0.9) | 317 (0.9) | <0.01 |
| **Prior myocardial infarction, n, (%)** | 2132 (1.6) | 468 (1.3) | 0.02 |
| **Onset-to-door time, median (IQR)** | 10.5 (6.8, 17.2) | 11.0 (6.9, 17.9) | 0.04 |
| **Tertiary hospital, n, (%)** | 66974 (48.8) | 19366 (54.2) | 0.11 |
| **Thrombolysis, n, (%)** | 874 (0.6) | 79 (0.2) | 0.06 |

Data are presented as median (interquartile range) or n (%). BMI, body mass index; IVT, intravenous thrombolysis; NIHSS, National Institutes of Health Stroke Scale; SMD, standardized mean difference; TIA, transient ischemic attack.

**Table S2. Baseline characteristics before propensity score matching.**

| **Variables** | **Non-IVT**  **(n=136283)** | **IVT**  **(n=874)** | **SMD** |
| --- | --- | --- | --- |
| **Age, years, median (IQR)** | 66.0 (57.0, 74.0) | 65.0 (56.0, 73.0) | 0.12 |
| **Male, n, (%)** | 88039 (64.6) | 581 (66.5) | 0.04 |
| **BMI, median (IQR)** | 23.9 (22.0, 25.8) | 23.4 (21.4, 25.7) | 0.09 |
| **History of smoking, n, (%)** | 48712 (35.7) | 340 (38.9) | 0.07 |
| **History of drinking, n, (%)** | 30124 (22.1) | 228 (26.1) | 0.09 |
| **Hypertension, n, (%)** | 86516 (63.5) | 499 (57.1) | 0.13 |
| **Diabetes mellitus, n, (%)** | 29401 (21.6) | 176 (20.1) | 0.04 |
| **Dyslipidemia, n, (%)** | 7084 (5.2) | 49 (5.6) | 0.02 |
| **Atrial fibrillation, n, (%)** | 4780 (3.5) | 49 (5.6) | 0.10 |
| **Prior TIA, n, (%)** | 1205 (0.9) | 11 (1.3) | 0.04 |
| **Prior ischemic stroke, n, (%)** | 35317 (25.9) | 186 (21.3) | 0.11 |
| **Prior peripheral vascular disease, n, (%)** | 1280 (0.9) | 5 (0.6) | 0.04 |
| **Prior myocardial infarction, n, (%)** | 2119 (1.6) | 13 (1.5) | 0.01 |
| **NIHSS score at admission, median (IQR)** | 3.0 (1.0, 4.0) | 4.0 (2.0, 8.0) | 0.52 |
| **Onset-to-door time, median (IQR)** | 10.6 (6.8,17.3) | 5.2 (4.5, 8.3) | 0.92 |
| **Tertiary hospital, n, (%)** | 66440 (48.8) | 534 (61.1) | 0.25 |

BMI, body mass index; TIA, Transient Ischemic Attack; NIHSS, National Institutes of Health Stroke Scale; SMD, standardized mean difference; ODT, Onset-to-Door time

**Table S3. Sensitivity analysis after multiple imputation for missing admission NIHSS scores.**

| **Outcome** | **Non-IVT (n=171,934)** | **IVT (n=953)** | **Adjusted estimate (95% CI)** | **P value** |
| --- | --- | --- | --- | --- |
| **Primary outcome** | | | | |
| **mRS 0–1 at discharge** | 44,678 (26.0) | 281 (29.5) | OR, 1.09 (1.06-1.12) | <0.001 |
| **Secondary discharge outcomes** | | | | |
| **mRS 0–2 at discharge** | 126,858 (73.9) | 623 (65.4) | OR, 1.04 (1.01-1.06) | 0.01 |
| **Independent ambulation at discharge** | 146,736 (85.5) | 726 (76.2) | OR, 1.01 (0.99-1.03) | 0.17 |
| **Discharge directly home** | 159,355 (92.7) | 846 (88.8) | OR, 0.99 (0.98-1.01) | 0.37 |
| **Length of hospital stay, days** | 10.00 (7.00–13.00) | 10.00 (7.00–14.00) | β, -0.59 (-0.95 to -0.23) | 0.001 |
| **Safety outcomes** | | | | |
| **Registry-recorded in-hospital ICH** | 717 (0.4) | 17 (1.8) | OR, 1.01 (1.01-1.02) | <0.001 |
| **In-hospital mortality** | 3,702 (2.2) | 57 (6.0) | OR, 1.00 (0.99-1.01) | 0.77 |
| **DAMA** | 10,210 (6.0) | 54 (5.7) | OR, 0.98 (0.96-0.99) | 0.002 |
| **In-hospital mortality or DAMA** | 12,625 (7.4) | 95 (10.0) | OR, 0.98 (0.97-1.00) | 0.04 |

ORs are reported for binary outcomes, and β coefficients are reported for length of hospital stay. β represents the adjusted mean difference in days for IVT versus non-IVT. Registry-recorded in-hospital ICH represented any intracranial hemorrhage recorded during hospitalization. Information on symptomatic status, ECASS/SITS classification, hemorrhage subtype, hemorrhage-related neurological deterioration, and fatal ICH was unavailable.

CI, confidence interval; DAMA, discharge against medical advice; ICH, intracranial hemorrhage; IVT, intravenous thrombolysis; mRS, modified Rankin Scale; NIHSS, National Institutes of Health Stroke Scale; OR, odds ratio.

**Table S4. Distribution of onset-to-needle time among patients treated with intravenous alteplase.**

| **Onset-to-needle time** | **IVT group (n=874)** |
| --- | --- |
| **ONT, hours, median (IQR)** | 6.05 (5.12-10.04) |
| **ONT, hours, range** | 4.50-23.98 |
| **4.5-<6 hours, n (%)** | 423 (48.4) |
| **6-<9 hours, n (%)** | 207 (23.7) |
| **9-<12 hours, n (%)** | 58 (6.6) |
| **12-24 hours, n (%)** | 186 (21.3) |

Data are presented as median (interquartile range), range, or n (%). ONT was available only for patients receiving intravenous thrombolysis and was therefore summarized descriptively within the IVT group. Percentages were calculated using the 874 IVT-treated patients as the denominator.

IQR, interquartile range; IVT, intravenous thrombolysis; ONT, onset-to-needle time.
